# Capecitabine in the routine first-line treatment of elderly patients with advanced colorectal cancer - results from a non-interventional observation study

**DOI:** 10.1186/s12885-016-2113-8

**Published:** 2016-02-10

**Authors:** Alexander Stein, Julia Quidde, Jan Klaus Schröder, Thomas Göhler, Barbara Tschechne, Annette-Rosel Valdix, Heinz-Gert Höffkes, Silke Schirrmacher-Memmel, Tim Wohlfarth, Axel Hinke, Andreas Engelen, Dirk Arnold

**Affiliations:** Department of Oncology, Hematology, BMT with section Pneumology, University Medical Center Hamburg-Eppendorf, Hamburg, Germany; Schwerpunktpraxis für Hämatologie und Onkologie, Mühlheim an der Ruhr, Germany; Onkozentrum Dresden/Freiberg, Dresden, Germany; Hämatologisch-onkologische Schwerpunktpraxis, Neustadt am Rübenberge, Germany; Onkologische Schwerpunktpraxis Schwerin, Schwerin, Germany; Medizinisches Versorgungszentrum, Fulda, Germany; Roche Pharma AG, Grenzach-Wyhlen, Germany; WiSP Research Institute, Langenfeld, Germany; CUF Hospitals Cancer Centre, Lisbon, Portugal

**Keywords:** Capecitabine, Metastatic colorectal cancer, Elderly patients

## Abstract

**Background:**

The purpose of this observational study was to evaluate feasibility, efficacy results and toxicity observations of capecitabine in routine first line treatment of patients with metastatic colorectal cancer, with particular regard of elderly patients (>75 years of age).

**Methods:**

Patients with colorectal cancer receiving capecitabine as part of their first-line treatment were recorded until detection of disease progression or up to a maximum of 12 cycles on standardized evaluation forms. Additional information on long-term outcomes, progression-free survival, and overall survival were retrieved at two follow-up time points. Obtained data were analyzed with regard to age up to 75 and >75 years of age. There were no specific requirements for patient selection and conduct of therapy, corresponding to the non-interventional nature of the study.

**Results:**

In total, 1249 evaluable patients were enrolled in Germany. The median age of the study population was 74 years (range: 21–99). Capecitabine-based combination was administered in 56 % of patients in the overall population. The median treatment duration was about 5 months. Severe toxicities occurred rarely without any difference regarding age groups. The most common hematological toxicity was anemia. Gastrointestinal side effects and hand-food-syndrome (HFS) were the most frequent non-hematologic toxicities. Overall response rate (ORR) was significantly higher in the patient group <=75 years compared to patients >75 years of age (38 vs. 32 %, p=0.019). Median progression free survival (PFS 9.7 vs. 8.2 months, p=0.00021) and overall survival (OS 31.0 vs. 22.6 months, p<0.0001) was decreased in elderly patients.

**Conclusion:**

Efficacy and tolerability of capecitabine treatment either as single drug or in various combination regimens, as proven in randomized studies, could be confirmed in a clinical routine setting. Patients older than 75 years may derive a relevant benefit by first line capecitabine-based treatment with good tolerability.

**Electronic supplementary material:**

The online version of this article (doi:10.1186/s12885-016-2113-8) contains supplementary material, which is available to authorized users.

## Background

While, for more than four decades, the treatment of colorectal cancer (CRC) consisted almost exclusively of the fluoropyrimidine 5-fluorouracil (5-FU) (eventually modulated by folinic acid or levamisole), the development of a plethora of new drugs since the 1990s has improved the therapeutic options in this indication. In addition to new agents with a classical cytotoxic mode of action, such as irinotecan and oxaliplatin, “targeted therapies”, such as monoclonal antibodies, tyrosine kinase inhibitors or fusion proteins directed against vascular endothelial growth factors (receptors) (VEGF(R)) or epidermal growth factor receptors (EGFR) have gained increasing importance [1, 2].

Administration of 5-FU, the main chemotherapy backbone in metastatic CRC (mCRC), is confined to either implanted venous access ports or in-patient treatment. Thus, the development of capecitabine, an oral fluoropyrimidine carbamate working as 5-FU prodrug, relevantly facilitated treatment for CRC patients. Capecitabine (Xeloda^®^) was licensed for the treatment of metastatic colorectal cancer mainly based on two large multicenter studies, both comparing the oral drug with a common 5-FU/leucovorin combination (Mayo regimen) [3, 4]. Following trials could establish the combination with oxaliplatin or irinotecan, and/or bevacizumab although particularly the combination of capecitabine and irinotecan has shown gastrointestinal side effects, which have to be closely monitored [5–10]. Currently, even chemotriplets with capecitabine, irinotecan and oxaliplatin with or without bevacizumab are being evaluated [11, 12]. Despite the long-standing application of capecitabine in the treatment of colorectal cancer, data in elderly patients are still limited.

The purpose of this non-interventional observation study was the acquisition of data on the routine usage of capecitabine, its efficacy and toxicity spectrum in routine clinical practice with particular focus on the benefit in patients >75 years of age.

## Methods

The project fulfilled the criteria of a non-interventional study according to the European Community and German legislation, and therefore required neither an ethical committee vote nor informed consent of the patients when the registry was started in 2004 [13]. In order to achieve a representative picture of capecitabine use in Germany, participation was offered to a large variety of office or hospital-based medical oncologists or gastroenterologists. Recruitment was limited to a pre-specified number of cases per investigator. The per patient-documentation fee was independent of the number of cycles documented. To ensure enrolment of a typical advanced CRC population, eligibility criteria were minimized to age ≥18 years, histologically confirmed advanced (metastatic or inoperable, locally advanced or recurrent) colorectal cancer without prior palliative treatment and eligibility for capecitabine treatment based on the summary of product characteristics. Treatment regimen (combination), diagnostics or frequency of examinations were scheduled by the respective treating hospital and office based clinicians. The applied dosage, treatment duration, cycle delays and/or therapy interruptions and eventually concomitant antineoplastic therapy were investigated in addition to demographic and baseline characteristics. Efficacy endpoints were overall response rate (ORR), PFS and OS. Tumor regression and progressive disease was recorded as the best response achieved, based on standard clinical procedures at the discretion of the investigators, without formal requirement of objective remission confirmation. Toxicity data were recorded after every second cycle (6 weeks), based on NCI CTC (National Cancer Institute Common Terminology Criteria for Adverse events) criteria (version 2). The detailed documentation was performed to a maximum of 12 cycles or until progression. Thereafter, key long-term data on overall survival and progression-free survival were retrieved by fax forms at two time points in 2010 and 2012.

The statistical methods were mainly descriptive. Most of the analyses presented were performed separately for the patient subgroups aged ≤75 and >75 years, respectively. Patients beyond 75 years of age are henceforward defined as “older” within this report. Capecitabine dosages were calculated individually, based on the reported absolute dose and the body surface of the patient. To compare baseline characteristics, and the response or toxicity rates in different patient groups, a Mantel-Haenszel test for trend or Fisher’s exact test was applied. PFS was defined from the first day of therapy with capecitabine to disease progression or death from any reason without prior progression. The PFS and OS curves were calculated according to the Kaplan-Meier method [14] and between-group comparisons were performed using the logrank test [15].

## Results

### Baseline characteristics

Between 2004 and 2011 altogether 1305 German patients were recruited of whom 1249 patients with advanced colorectal cancer were eligible for the evaluation. The majority of patients (60 %) were recruited in the second half of the period (between 2008 and 2011). Table 1 provides a description of the study population and their baseline tumor characteristics. A considerable number of older patients participated in this non-interventional trial shown by a median age of the study population of 74 years. At the start of therapy the ECOG (Eastern Cooperative Oncology Group) performance status was not impaired (grade 0 in 28 %) or only slightly reduced (grade 1 in 52 %) in the majority of patients. Performance status in older patients was limited compared to patients up to the age of 75 years (*p* <0.0001). In 47 % of patients locoregional disease was present at study entry. Synchronous metastatic disease was reported in 646 patients. In the 541 patients with metachronous metastases the median relapse-free interval, defined as the time between first diagnosis of the disease and first detection of metastases was 1.6 years. Nearly two thirds (64 %) of the patients suffered from liver metastases and 29 % from lung involvement with the latter occurring significantly less frequently in older patients (*p* = 0.0057). Bone and central nervous system (CNS) lesions, pleural effusion and ascites occured rarely with an incidence of below 5 % each. Almost all patients (89 %) received initial surgery of their primary tumor, interestingly with no differences regarding age. Radiotherapy was applied in 15 % of the patients with a significantly decreasing proportion in older patients (*p* <0.0001). Overall 252 (30 %) patients received prior chemotherapy for non-metastatic disease (neo-/adjuvant). Chemotherapeutic pretreatment was more common in younger patients with a continuous and significantly decreasing percentage with age (37 % in patients younger than 76 years compared to 19 % in patients beyond 75 years; *p* <0.0001). Median time between the end of prior chemotherapy and initiation of systemic treatment for advanced disease and thus beginning of the observation period was 1 year. In 27 % of these patients the interval was longer than 2 years.

**Table 1:** Patient and tumor characteristics

No. of patients (%)	≤75 years 711 (57 %)	>75 years 538 (43 %)	Total 1249 (100 %)
Age, median (range), years			74 (21–99)
Sex, no. of patients (%)			
Male	418 (59 %)	266 (49 %)	684 (55 %)
Female	293 (41 %)	272 (51 %)	565 (45 %)
ECOG performance, no. of patients (%)			
0	224 (32 %)	111 (22 %)	335 (28 %)
1	350 (50 %)	281 (55 %)	631 (52 %)
2	107 (15 %)	105 (20 %)	212 (18 %)
3	12 (2 %)	18 (3 %)	30 (2 %)
4	1 (0 %)	0 (0 %)	1 (0 %)
Grading, no. of patients (%)			
G0/G1	23 (3 %)	15 (3 %)	38 (4 %)
G2	413 (62 %)	323 (66 %)	736 (64 %)
G3	201 (30 %)	129 (26 %)	330 (29 %)
GX	27 (4 %)	21 (4 %)	48 (4 %)
Disease site at entry, no. of patients (%)^a^			
Local	325 (46 %)	259 (48 %)	584 (47 %)
Liver	453 (64 %)	352 (65 %)	805 (64 %)
Lung	230 (32 %)	135 (25 %)	365 (29 %)
Bone	30 (4 %)	24 (4 %)	54 (4 %)
CNS	6 (1 %)	2 (0 %)	8 (1 %)
Pleural effusion	16 (2 %)	9 (2 %)	25 (2 %)
Ascites	21 (3 %)	24 (4 %)	45 (4 %)
Other	151 (21 %)	95 (18 %)	246 (20 %)
Relapse-free interval^b^, median, years n = 541 patients^c^	1.8	1.5	1.6
M1 at initial diagnosis, no. of patients (%) n = 1066 patients^c^	367 (59 %)	279 (63 %)	646 (61 %)
Previous surgery, no. of patients (%) n = 1248 patients^c^	626 (88 %)	480 (89 %)	1106 (89 %)
Previous radiotherapy, no. of patients (%) n = 1239 patients^c^	137 (19 %)	53 (10 %)	190 (15 %)
Previous (neo) adjuvant chemo- therapy, no. of patients (%) n = 847 patients^c^	187 (37 %)	65 (19 %)	252 (30 %)

^a^the choice of multiple categories was possible

^b^patients with metachronous metastases only

^c^evaluable patients for the respective parameter

### Treatment

580 patients (46 %) received single agent capecitabine, the other 668 patients (54 %) were treated with capecitabine in combination with one (39 %) or two (15 %) additional drugs (Table 2). The most frequently used concomitant drugs were oxaliplatin (43 %), bevacizumab (36 %) and irinotecan (17 %). Only 27 patients (4 %) received capecitabine in combination with EGFR antibodies. Single agent capecitabine was predominantly administered in the group of patients aged over 75 years (65 %) and in almost three out of four patients older than 85 years (74 %). Notably, the rates of oxaliplatin-based combinations were significantly lower in older patients (only 15 % of patients >75 years; *p* <0.0001).

**Table 2:** Type of regimens

Regimens	≤ 75 years	>75 years	Total
n	710	538	1248
Capecitabine (mono)	232 (33 %)	348 (65 %)	580 (46 %)
Capecitabine + 1 cytostatic/antibody	312 (44 %)	169 (31 %)	481 (39 %)
Capecitabine + 2 cytostatics/antibodies	166 (23 %)	21 (4 %)	187 (15 %)
Capecitabine + Irinotecan	31 (4 %)	22 (4 %)	53 (4 %)
Capecitabine + Oxaliplatin	186 (26 %)	82 (15 %)	268 (21 %)
Capecitabine + antibody	87 (12 %)	64 (12 %)	151 (12 %)
Capecitabine + Irinotecan + antibody	80 (11 %)	7 (1 %)	87 (7 %)
Capecitabine + Oxaliplatin + antibody	80 (11 %)	10 (2 %)	90 (7 %)
Capecitabine + other combination	14 (2 %)	5 (1 %)	19 (2 %)

The median treatment duration with capecitabine was 5.3 months. Only 8 % of patients received capecitabine for more than 10 months. In general, treatment duration tended to be independent of patients’ age with medians of 5.3 months in both age cohorts. The overall median daily dose of capecitabine was 1727 mg/m^2^ with only very small difference between the two age groups (1744 mg/m^2^ for younger and 1702 mg/m^2^ for elderly patients). For the monotherapy the observed median dose of capecitabine was 1838 mg/m^2^. As to be expected, the dosage was lower when combination chemotherapy was given (1635 mg/m^2^), with medians between 1300 and 1777 mg/m^2^ depending on the type of combination regimen (Additional file 1: Table S1).

Treatment courses were delayed in only 13 % of all cycles, but nevertheless occurred at least once in 54 % of patients. Capecitabine dose reductions were reported in 22 % of all cycles and 45 % of all patients, respectively. In terms of treatment discontinuation, no distinct difference could be observed regarding age whereas dose reduction occurred more often in younger patients, likely related to the higher rate of combination regimen (*p* = 0.040).

In 663 patients (53 %) the observation was terminated according to the observation plan, either due to progressive disease (58 %) or due to reaching the maximum documentation period of 12 cycles (42 %). In the remaining 580 patients (47 %), reasons for a premature termination of the treatment were side effects (23 %), patient’s wish/non-compliance (15 %) and death due to the underlying disease (11 %). Other reasons (52 %) mainly were an a priori planned number of cycles of less than 12 or the achievement of a good response.

### Toxicity

Table 3 shows the hematological and non-hematological adverse events according to age groups (split by median age) and combination vs. monotherapy. The NCI toxicity grade per patient was pre-specified in the observation forms. Regardless of age, severe hematological and non-hematological toxicities (grade 3 or 4) occurred only rarely, despite the high rate of combination regimens applied (54 % of patients). The most common toxicities were gastrointestinal disorders like nausea, diarrhea or vomiting, as well as hand-foot-syndrome (HFS) occurring in 42 % of the patients. Expectedly, oxaliplatin-based compared to irinotecan-based regimen showed different rates of all grade thrombocytopenia (33 % vs. 11 %), diarrhea (40 % vs. 49 %), alopecia (21 % vs. 41 %) and neurological disorders (21 % vs. 11 %), respectively. The proportion of patients affected by HFS increased with the duration of the treatment. In cycles 1 and 2, HFS occurred in only 15 % of the patients, increasing to 27 % in cycles 3 and 4, 32 % in cycles 5 and 6 and finally 39 % in cycles 7 and 8. Despite similar median daily dosage of capecitabine, all grade HFS was more frequent in younger patients (46 %) compared to only 37 % in patients older than 75 years (*p* = 0.0014). However, the rates of grade 3/4 HFS were similar in both age groups (Table 3).

**Table 3:** Hematological and non-hematological toxicity (maximum pre patient and type)

Toxicity	NCI grade 3/4			
	≤ 75 years	>75 years	Single agent	Combination
Anemia	21 (4 %)	10 (2 %)	22 (4 %)	9 (2 %)
Leukopenia	10 (2 %)	4 (1 %)	1 (0 %)	13 (2 %)
Neutropenia	8 (2 %)	7 (2 %)	2 (0 %)	13 (2 %)
Thrombopenia	12 (2 %)	5 (1 %)	6 (2 %)	11 (2 %)
Nausea	15 (2 %)	9 (2 %)	11 (2 %)	13 (2 %)
Vomiting	8 (2 %)	5 (1 %)	4 (1 %)	9 (2 %)
Diarrhea	24 (4 %)	13 (2 %)	12 (2 %)	26 (4 %)
Mucositis/ stomatitis	4 (1 %)	4 (1 %)	6 (1 %)	3 (1 %)
Bilirubin elevation	16 (3 %)	20 (4 %)	19 (4 %)	27 (5 %)
Neuropathy-motor	13 (2 %)	18 (3 %)	19 (4 %)	14 (3 %)
Hand-foot-syndrome	18 (3 %)	19 (4 %)	21 (4 %)	17 (3 %)
Fever	0 (0 %)	1 (0 %)	1 (0 %)	0 (0 %)
Pain	16 (3 %)	15 (3 %)	13 (2 %)	19 (4 %)
Other	28 (5 %)	19 (3 %)	15 (3 %)	34 (6 %)

### Treatment efficacy

**Fig. 1:**
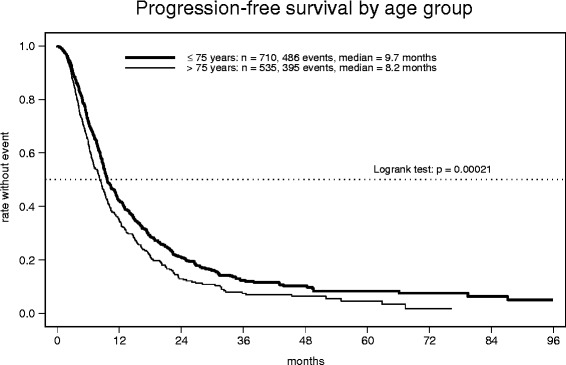
Progression-free survival by age group

**Fig. 2:**
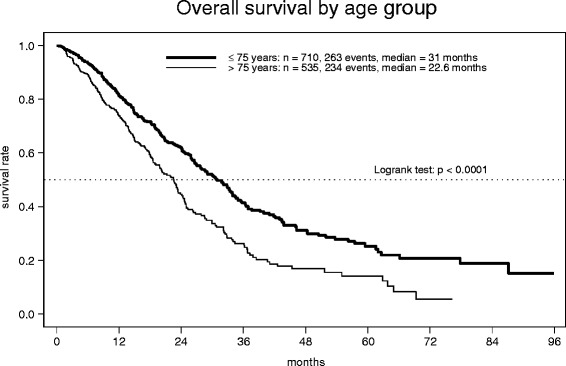
Overall survival by age group

1237 patients were evaluable for objective tumor response (Table 4). 35 % of these achieved an objective remission (complete in 6 % and partial in 29 %), with rates decreasing from 38 % to 32 % in the respective age cohorts (*p* = 0.019). For the evaluation of the parameters PFS and OS 1245 patients could be included. The Kaplan-Meier estimate of PFS, based on 881 events in 1245 patients (71 %) showed a median of 9.2 months for the whole observation group (Additional file 2: Figure S1). Median PFS in elderly patients was significantly decreased with 8.2 months, compared to 9.7 months in patients up to 75 years of age (*p* = 0.00021, HR = 1.29, 95 % CI 1.13–1.47) (Fig. 1). In comparison to single drug capecitabine with a median PFS of 8.3 months, a non-significant difference towards an improved PFS with median 9.8 months could be observed for the combination regimen (*p* = 0.063, HR = 0.88, 95 % CI 0.77–1.01). For 497 (40 %) out of 1245 patients the date of death was documented, resulting in an overall median OS of 26.1 months. In elderly patients OS was significantly decreased (median 22.6 months), compared to 31.0 months in patients up to 75 years of age (*p* < 0.0001, HR = 1.61, 95 % CI 1.35–1.92) (Fig. 2). In contrast, the comparison of regimen types (single agent vs. combination) showed no differences in terms of OS (25.9 vs. 26.1 months, *p* = 0.71, HR = 0.97, 95 % CI 0.81–1.15).

**Table 4:** Tumor response

	≤ 75 years	>75 years	Total
Full analysis set (no. of patients (%))			
n	704	533	1237
CR	54 (8 %)	24 (5 %)	78 (6 %)
PR	215 (31 %)	145 (27 %)	360 (29 %)
SD	232 (33 %)	165 (31 %)	397 (32 %)
PD	98 (14 %)	94 (18 %)	192 (16 %)
Insufficient assessment	105 (15 %)	105 (20 %)	210 (17 %)

Patients suffering from HFS of any grade during therapy had a higher response rate (43 %) than patients without this symptom (31 %) (*p* < 0.0001). Moreover, PFS and OS were significantly prolonged in patients who experienced HFS. The median PFS was 10.6 months vs. 8.2 months (*p* <0.0001, HR: 0.86 [95 % CI: 0.81–0.93]) and the median OS to 28.8 months vs. 24.2 months (*p* = 0.00038, HR: 0.85 [95 % CI: 0.77–0.93]) in patients suffering from HFS CTC grade 1–3 compared to those without this symptom.

A similar favorable trend could also be observed in patients who received a capecitabine dose reduction during therapy, likely associated with the occurrence of HFS. The median PFS was 10 months vs. 8.3 months (*p* = 0.0009, HR: 0.89 [95 % CI: 0.83–0.95]) and the median OS was 28.6 months vs. 23.8 months (*p* = 0.0026, HR: 0.87 [95 % CI: 0.80–0.95]) in patients who received dose reduction compared to those who did not.

## Discussion

The efficacy observed in this observational study is in the same range as previously reported from randomized trials. The reported ORR of 35 % mirrors the results obtained in previous single agent or combination trials, e.g., with oxaliplatin or bevacizumab [6, 9, 16]. The median PFS results observed compare favorably to prior trials, both for the overall as well as in the different subgroups regarding age and combination with other agents. However, these differences may be explained, at least in part, by the presence of an observation bias due to a less stringent re-staging schedule in our routine observation study compared to the randomized trials. With respect to median overall survival, our results compare quite favorably to those of the early single drug registration studies and capecitabine-based doublets [6, 16, 17]. The median OS of 26.1 months observed in our cohort is likely influenced by the observed shift in median OS during the last decades due to the availability of new agents and the increasing integration of locally ablative procedures. In comparison to former registries in first line mCRC e.g., BEAT or BRiTE recruited between 2004 and 2006 showing a median OS of up to 23 months the main recruitment period of this study (60 % of patients recruited between 2008 and 2011) was later and thus reflecting more the current developments of a median OS of about 30 months achieved in current first line trials [18–21]. Furthermore, the patient population seemed to have a favorable prognosis, in terms of a rather high rate of a good ECOG PS (0 or 1) despite the median age of 74 years and the resection of their primary tumor in the vast majority of patients [22]. Besides increased ORR (27 vs. 42 %) and median PFS (8.3 vs. 9.8 months, *p* = 0.06) comparing single agent and combination regimen, median OS was similar (25.9 vs. 26.1 months, *p* = 0.71). Combination regimen were more often applied in younger patients (77 % of patients ≤65 years compared to 26 % of patients >85 years) with likely more aggressive disease (e.g., higher rate of poorly differentiated tumors, multiple metastastic sites).

Regarding toxicity, capecitabine-based regimen can be administered without major complications in most patients. The rate of severe toxicities (grade 3 or 4) was below 5 % with respect to all NCI CTC categories in this observational study. Hand-foot-syndrome, which proved to be the dose-limiting toxicity of single drug capecitabine, was reported in somewhat less than half of our patients, but proved to be manageable, with only 3 % of the patients suffering from serious symptoms.

The treatment efficacy was decreased in the elderly subgroup (>75 years), but still remained on a high level with an ORR of 32 %, median PFS of 8.2 months and OS of 22.6 months. Therefore, the overall efficacy results of this large group of elderly patients is within the range as previously reported, considering the relevant number of elderly patients receiving combination treatment [16, 23, 24]. Moreover, tolerability did not seem to be limited in elderly patients with regard to grade 3/4 toxicities. During the last decade treatment approach in elderly patients has significantly changed, whereas early trials evaluated only single agent fluoropyrimidines, combination regimen are currently more frequently administered [25–27]. Elderly patients with a good performance status eligible for clinical phase 3 trials seem to derive a relevant benefit by the addition of further agents as shown in different subgroup analyses [28]. Randomized trials have furthermore established the relevant benefit and tolerability of combination regimen in elderly patients [16, 23, 29]. In order to stratify elderly patients to the different treatment intensities comprehensive geriatric assessment should be applied and is recommended by current guidelines [30].

Besides, capecitabine has been shown to be well tolerated and efficacious in elderly patients in the adjuvant setting as single agent compared to bolus 5FU/LV [31]. In addition, capecitabine (or infusional 5-FU) seems to be the favorable combination partner if an oxaliplatin-based adjuvant treatment is chosen. In contrast to prior data including bolus 5FU based regimen (FLOX), Haller and colleagues recently demonstrated an attenuated but sustained DFS and OS benefit in elderly patients with a modern fluoropyrimidine schedule in combination with oxaliplatin (CAPOX or FOLFOX) [32–34].

Further oral fluoropyrimidines (S-1 or UFT) have been studied in localized or metastatic CRC, showing tolerability and efficacy as single agent or in different combinations (e.g., oxaliplatin – SOX/TEGAFOX regimen or irinotecan – IRIS/TEGAFIRI) without any apparent interaction with age [35–39]. Similar to capecitabine, these oral fluoropyrimidines can be safely combined with bevacizumab in elderly patients [24, 40]. Recently, TAS 102 has shown a significant survival benefit in heavily pretreated mCRC patients with good tolerability and similar OS benefit for patients ≤/>65 years [41].

Dose reductions of capecitabine during treatment does not lead to a poorer long-term outcome, as previously reported [42, 43]. However, due to the observational nature of our study, it is not possible to differentiate the effects of dose modifications from the association with HFS, the development of which seems to be a strong favorable prognostic factor by itself. The occurrence of toxicities like HFS and the consecutive dose reductions likely are clinical markers of the individual effective dosage of capecitabine.

The general characteristics of a non-interventional study focusing on a specific drug inevitably lead to major limitations, particularly in regard of bias in terms of patient selection. An intention-to-treat analysis, was performed with no documented, eligible patient excluded. However, as inclusion of patients into the observational study was not entirely under our control, we cannot completely rule out, that in individual patients with a very short treatment course (e.g., due to early death), the record file was not sent to the documentation centre (although this was clearly not intended or suggested). Moreover, we cannot control or adjust for any selection effect that is associated with the decision to use infusional 5-FU instead of the oral alternative. Possibly, high-risk patients with an immediate need for tumor shrinkage may be underrepresented. Moreover, decisions on treatment intensity will likewise be depending on patients’ age and, thus, subgroup analyses based on these characteristics are biased by this interdependence. Validity and completeness of tumor response and toxicity data is typically lower compared to randomized controlled trials.

## Conclusions

Based on the shown efficacy and tolerability, capecitabine is a valid option for the treatment of colorectal cancer without any unequivocally apparent age limit.

## Additional files

Additional file 1: Table S1. Mean daily Capecitabine doses (mg/m^2^) in terms of regimen types. (DOC 30 kb) Additional file 2: Figure S1. Progression-free survival; n = number of patients. (PPT 81 kb)

## Abbreviations

5-FU: 5-Fluorouracil
BEAT: Bevacizumab Expanded Access Trial
BRiTE: Bevacizumab Regimens: Investigation of Treatment Effects and Safety
CNS: central nervous system
CRC: colorectal cancer
ECOG PS: Eastern Cooperative Oncology Group Performance Status
EGFR: epidermal growth factor receptor
HFS: hand-foot-syndrome
mCRC: metastatic colorectal cancer
NCI CTC: National Cancer Institute Common Terminology Criteria for Adverse events
ORR: overall response rate
OS: overall survival
PFS: progression free survival
VEGF(R): vascular endothelial growth factor (receptor)
