# Supplementary material for: Capecitabine in the routine first-line treatment of elderly patients with advanced colorectal cancer - results from a non-interventional observation study
Source: BMC Cancer. 2016 Feb 10;16:82. doi: 10.1186/s12885-016-2113-8 (PMC4750193; doi:10.1186/s12885-016-2113-8)
Supplement: Additional file 1: Table S1. — Mean daily Capecitabine doses (mg/m2) in terms of regimen types. (DOC 30 kb) [file 12885_2016_2113_MOESM1_ESM.doc]

## Table S1. Mean daily Capecitabine doses (mg/m²) in terms of regimen types

|  | Capecitabine mono | Capecitabine + antibody | Capecitabine + Irinotecan | Capecitabine +Irinotecan +antibody | Capecitabine + Oxaliplatin | Capecitabine + Oxaliplatin + antibody | Capecitabine + other | Total |
| --- | --- | --- | --- | --- | --- | --- | --- | --- |
| Evaluable patients | |  |  |  |  |  |  |  |
| N | 573 | 151 | 53 | 86 | 267 | 89 | 19 | 1238 |
| Mean ± SD | 1803.8± 451.8 | 1698.9± 405.9 | 1526.8± 378.4 | 1476.3± 351.6 | 1619± 355.3 | 1645.9± 375.3 | 1375.1± 448.5 | 1698.6± 426.6 |
| Median | 1838.8 | 1719.4 | 1549 | 1550.8 | 1696.1 | 1776.6 | 1300.4 | 1726.8 |
| Quartile | 1466.8- 2144.6 | 1391.6- 1968.6 | 1265.4- 1795.7 | 1263.1- 1663.5 | 1345.2- 1922.1 | 1393.3- 1927.7 | 993.1- 1693.2 | 1387.4- 1995.3 |
| Range | 571.2- 2634.4 | 879- 2578.9 | 789.9- 2478.9 | 721.1- 2860.1 | 795- 2340.5 | 791.3- 2403 | 711- 2354.1 | 571.2- 2860.1 |
