# Supplementary figures and images for: Capecitabine in the routine first-line treatment of elderly patients with advanced colorectal cancer - results from a non-interventional observation study
Source: BMC Cancer. 2016 Feb 10;16:82. doi: 10.1186/s12885-016-2113-8 (PMC4750193; doi:10.1186/s12885-016-2113-8)

## Slide 1
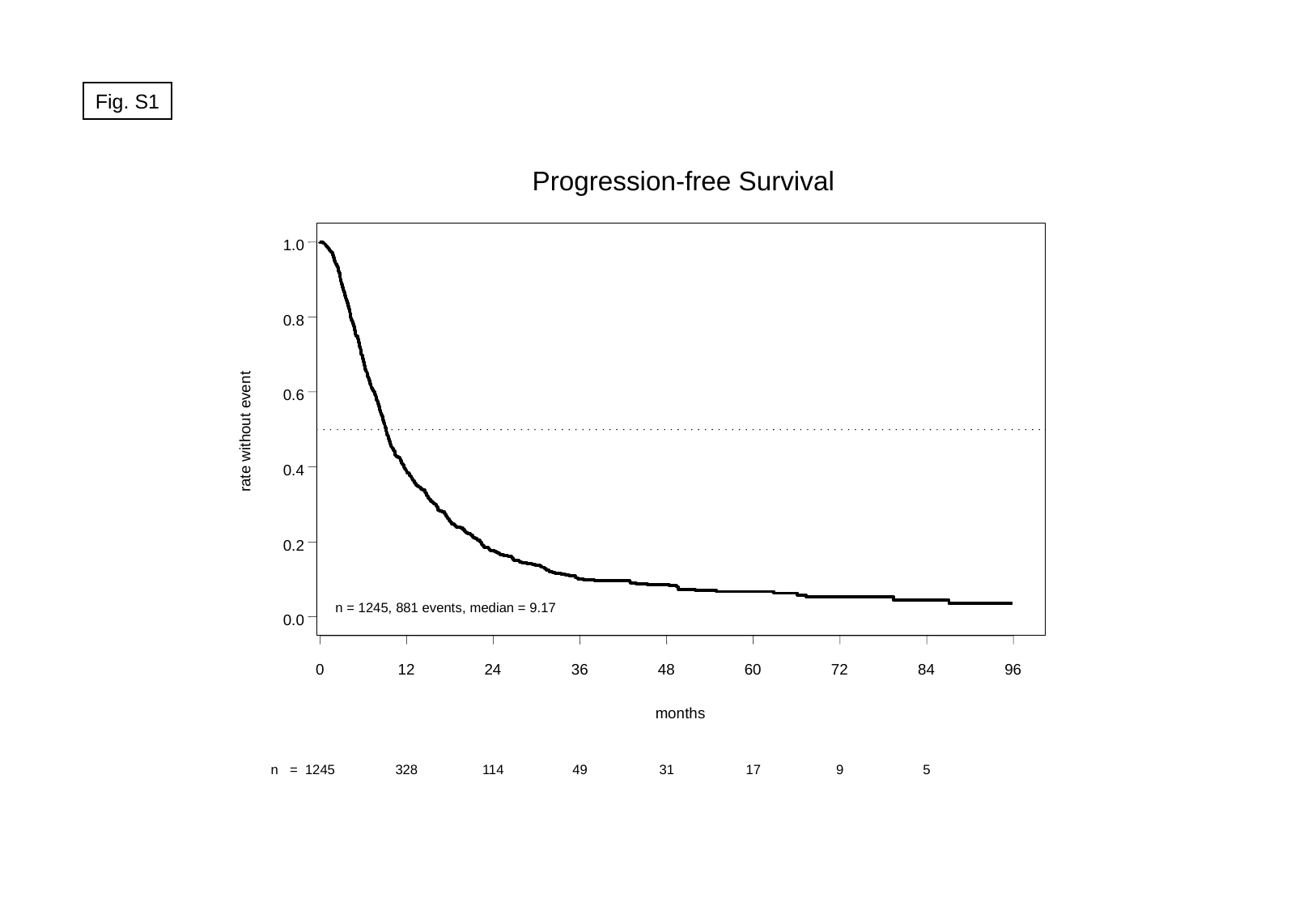

Fig. S1
Progression-free Survival
1.0
0.8
0.6
rate without event
0.4
0.2
n = 1245, 881 events, median = 9.17
0.0
0
12
24
36
48
60
72
84
96
months
n =
1245
328
114
49
31
17
9
5

Supplement: Additional file 2: Figure S1. — Progression-free survival; n = number of patients. (PPT 81 kb) [file 12885_2016_2113_MOESM2_ESM.ppt]
